# Bone microarchitecture and volumetric bone density impairment in young male adults with childhood-onset growth hormone deficiency

**DOI:** 10.1530/EJE-18-0711

**Published:** 2018-11-26

**Authors:** Hongbo Yang, Kemin Yan, Xu Yuping, Qi Zhang, Linjie Wang, Fengying Gong, Huijuan Zhu, Weibo Xia, Hui Pan

**Affiliations:** 1 Department of Endocrinology, Key Laboratory of Endocrinology of National Health Commission, The Translational Medicine Center of PUMCH; 2 Department of Clinical Laboratory, Peking Union Medical College Hospital, Chinese Academy of Medical Sciences and Peking Union Medical College, Beijing, China

## Abstract

**Context:**

Adult growth hormone deficiency (AGHD) is characterized by low bone density and increased risk of fracture. Bone microarchitecture is insufficiently evaluated in patients with childhood-onset AGHD (CO AGHD).

**Objective:**

To assess volumetric bone density (vBMD) and bone microarchitecture in CO AGHD in early adulthood after cessation of recombinant growth hormone (rhGH) treatment.

**Design and subjects:**

Case–control study in a major academic medical center in Beijing, including 20 young male adults with CO AGHD and 30 age- and weight-matched non-athletic healthy men. High-resolution peripheral quantitative computerized tomography (HR-pQCT) of distal radius and tibia was performed.

**Outcomes:**

The main outcomes were vBMD and morphometry parameters from HR-pQCT.

**Results:**

Compared with healthy controls, CO AGHD group had significantly decreased insulin-like growth factor 1 (IGF-1) level and IGF-1 SDS (*P* < 0.001). β-CTX and alkaline phosphatase levels in CO AGHD group were significantly increased (*P* < 0.001). CO AGHD group had significantly decreased total vBMD, cortical vBMD, trabecular vBMD, cortical area, cortical thickness as well as trabecular thickness and trabecular bone volume fraction of both tibia and radius (*P* < 0.001). CO AGHD patients had an 8.4 kg decrease in grip strength and a significant decrease in creatinine levels (*P* = 0.001). At both tibia and radius, by finite element analysis, bone stiffness and failure load of the CO AGHD patients were significantly decreased (*P* < 0.001). After adjusting for age, BMI and serum levels of testosterone and free thyroxin, serum IGF-1 level was a positive predictor for total vBMD, cortical vBMD, cortical area, trabecular vBMD, bone stiffness and failure load of both tibia and distal radius in all subjects.

**Conclusions:**

Young adult male patients with childhood-onset adult growth hormone deficiency who are no longer receiving growth hormone replacement have prominently impaired volumetric bone density and bone microarchitecture and lower estimated bone strength.

## Introduction

Adult growth hormone deficiency (AGHD) is an uncommon but debilitating disorder characterized by low bone mineral density, sarcopenia, increased risk of metabolic syndrome and decreased quality of life (1). Growth hormone promotes linear growth and healthy development in childhood and plays crucial roles in normal body function in adulthood, including maintenance of bone strength, muscle volume and other metabolic parameters. Since recombinant human growth hormone (rhGH) became available in 1985, it was reported to be effective in improving metabolic parameters, bone mineral density and quality of life in AGHD patients (2).

Dual-energy X-ray absorptiometry (DXA) at the hip and lumbar spine is widely used to evaluate skeletal situation in AGHD patients before and after rhGH replacement therapy (3, 4, 5). However, DXA only evaluates 2D areal bone mineral density (aBMD) and does not provide details of microarchitecture of cortical and cancellous bones. Besides, there might be false increases of aBMD due to bone hyperplasia, spinal degeneration and aortic calcification in lumbar spine DXA. Thus, DXA is lacking sensitivity and specificity in predicting fracture risks (6). High-resolution peripheral quantitative CT (HR-pQCT) allows noninvasive assessment of bone microstructure and volumetric bone mineral density (vBMD) at peripheral sites at high resolution (82 μm isotropic voxel size) and with relatively low radiation exposures (3–5 μSv) (7). Recently, bone microarchitecture was assessed by HR-pQCT in 16 male patients with unreplaced adulthood-onset AGHD (AO AGHD) (8). In that report, the mean age of AO AGHD patients was 47.4 years and the age of diagnosis was 37.1 years. No bone microarchitecture deficiency was reported in this series of patients.

**Figure 1:**
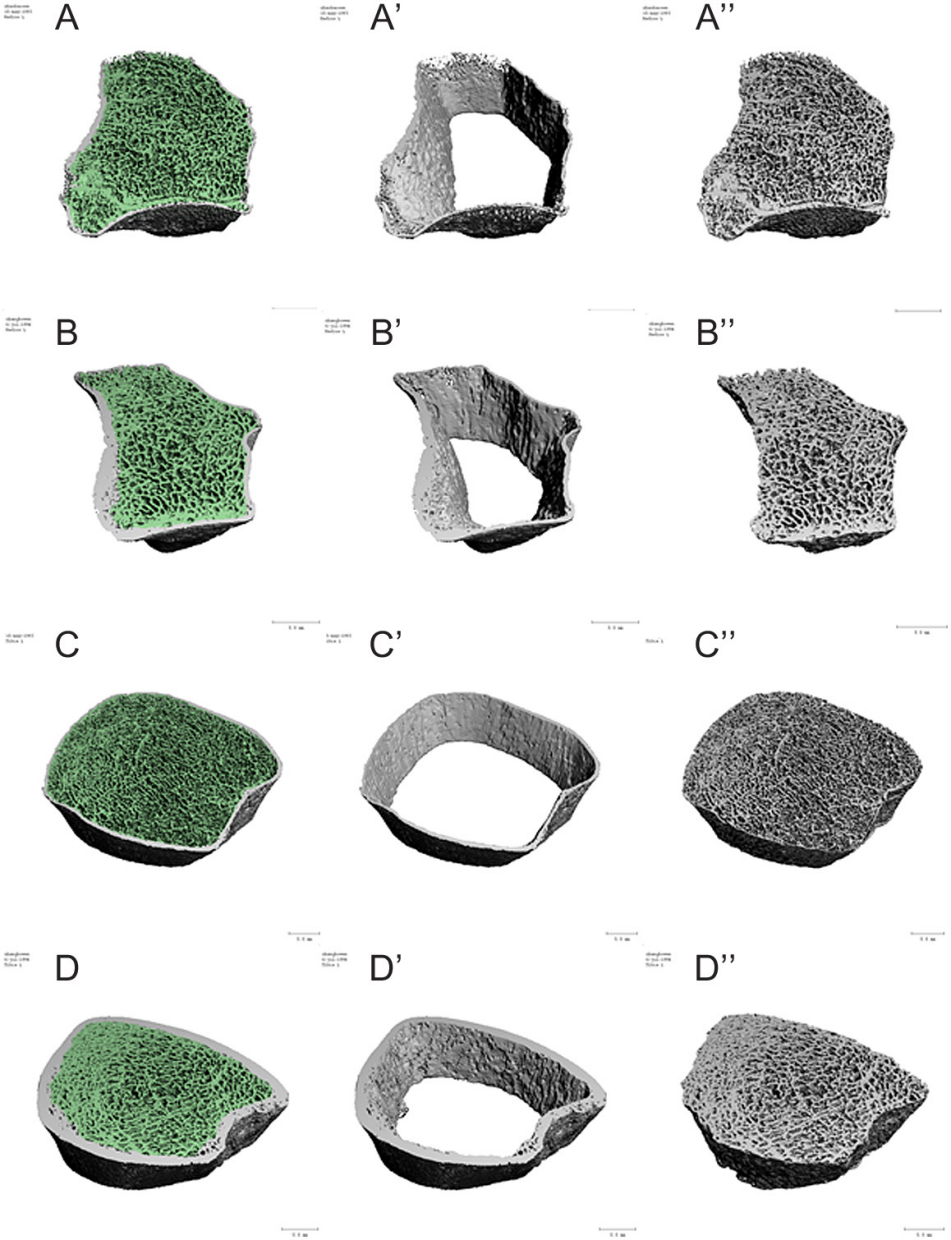
Representative images depicting bone microarchitecture of the non-dominant radius and distal tibia of a CO AGHD patient and a healthy control. Left panel show the whole bone microarchitecture the non-dominant radius in CO AGHD patient (A) and healthy control (B), distal tibia in CO AGHD patient (C) and healthy control (D). Middle panel highlights decreased cortical thickness in both radius and distal tibia in a patient with CO AGHD (A′ and C′) compared with healthy control (B′ and D′). Right panel shows deficient trabecular bone in both radius and distal tibia in a CO AGHD patient (A″ and C″) compared with healthy control (B″ and D″).

As we know, peak bone mass is the maximum amount of bone during individual’s life. It typically occurs in the early 20s in females and late 20s in males. Growth hormone and gonadosteroids play synergistic roles in peak bone mass. Most adolescents with multiple pituitary hormone deficiency (MPHD) will stop rhGH treatment after completion of linear growth but will sustain replacement therapy of other hormones (9). At present, no data are available for vBMD and bone microarchitecture in AGHD patients due to childhood-onset MPHD after cessation of rhGH treatment in early adulthood.

In this cross-sectional study, we assessed the vBMD, bone microarchitecture and estimated bone strength of young male adults with CO AGHD after cessation of rhGH replacement therapy. Female subjects were not enrolled to avoid potential gender-dimorphic variations in bone microarchitecture. The relationship between insulin-like growth factor 1 (IGF-1) levels and changes of vBMD, microarchitecture and estimated bone strength was also evaluated.

## Subjects and methods

### Subjects

A total of 20 consecutive male patients with CO AGHD were enrolled in this cross-sectional study from April 2017 to May 2018 in our GHD clinic at Peking Union Medical College Hospital (10). The diagnosis of AGHD was according to the criteria of American Endocrine Society Clinical Practice Guideline (11). Six patients underwent insulin tolerance test and peak value of GH was less than 3 ng/mL in all these patients. The diagnosis was confirmed in the other 14 patients since IGF-1 levels were below the age-adjusted normal range and there were deficiencies in three or more pituitary axes at the same time. All patients had stopped rhGH replacement therapy since attainment of final height. A total of 30 non-athletic healthy male were recruited from general population as controls. Approval from the Institutional Review Board of Peking Union Medical College Hospital was obtained for this study. Written informed consent was obtained from all participants. All data were de-identified before analysis.

### Anthropometrics

Height, weight, waist circumference and hip circumference of the subjects were measured by standard protocols in the early morning with light clothes. BMI was calculated as weight (kg) divided by height (m) squared.

### Bioimpedance assessments and body composition

Bioelectric impedance assessments (BIAs) by body composition analyzer (Tanita TBF-410, Japan) were performed on each of the participants according to manufacturer’s instructions. Measurements were taken in the morning after overnight fasting. Fat-free mass (FFM), fat-free mass percentage (FFM%), fat mass (FM), FM percentage (FM%) and total body water (TBW) were obtained according to the body composition model. FFM derived from BIA has been validated previously against DXA (12). The FFM index and FM index were calculated with formula by Van Itallie *et al*., namely FFMI = FFM/height^2^ (kg/m^2^) and FMI = fat mas/height^2^ (kg/m^2^) (13).

### Muscle strength

Grip strength was measured using a hand dynamometer (Jamar Plus+, Sammons Preston, Rolyon, Bolingbrook, IL, USA) on the non-dominant hand. All participants performed three trials of a maximum effort squeeze contracting for 5 s and the maximum values were recorded.

### High-resolution peripheral computed tomography (HR-pQCT) and finite element analysis

High-resolution images of the non-dominant distal radius and distal tibia were obtained by HR-pQCT scan (XtremeCT II scanner, ScancoMedical, Brüttisellen, Switzerland). vBMD and bone microarchitecture at the distal tibia and radius were investigated using a previously described protocol (14). All participants were scanned with the standard human *in vivo* scanning protocol (60 kVp, 1000 μA, 100 ms integration time). In short, when a scout scan was finished, reference lines were placed at the distal end plates for both the radius and tibia. Each scan comprised 110 slices, corresponding to a 10.2 mm scan area, with a nominal isotropic resolution of 82 μm carried out at the standard location 9.0 mm (radius) and 22.0 mm (tibia) proximal to the reference line. All scans were finished by a specified technologist and analyzed according to the standard manufacturer’s method (15). Scans were graded for motion artifacts as described previously (16).

Bone microarchitecture parameters, including total area (Tt.Ar), trabecular area (Tb.Ar), cortical area (Ct.Ar), cortical perimeter (Ct. Pm), total volume bone mineral density (Tr.vBMD), trabecular vBMD (Tb.vBMD), cortical vBMD (Ct.vBMD), trabecular separation (Tb. Sp), inhomogeneity of network (Tb.1/N.SD), cortical thickness (Ct.Th) and cortical porosity (Ct.Po), were obtained by the standard morphologic analysis using semi-automated software (15). The finite element analysis protocol has been previously described (8, 17). Using dedicated software, HR-pQCT images of the radius and tibia were converted into finite element models to estimate bone stiffness (N/mm). Failure load (N) was calculated using the Pistoia criterion (18).

### Biochemical measurements

The blood samples were obtained on the day of bone assessment. Liver function, kidney function, HbA1c, serum calcium, serum phosphate, alkaline phosphatase (ALP), 25-hydroxyvitamin D (25 OHD), parathyroid hormone (PTH), β-C-terminal telopeptide region of collagen type 1 (β-CTX) and hormonal evaluation were all tested in the department of clinical laboratory of Peking Union Medical College Hospital by standard methods. The serum IGF-1 level was measured with a fully automated two-site, solid-phase, chemiluminescent enzyme immunometric assay (Immulite 2000, Siemens Healthcare Diagnostics).

### Statistical analysis

Data are presented as the mean ± standard deviation (s.d.). The independent-samples *t* test was used for data analysis between the two groups. Skewed data were in-transformed before *t* test. The Mann–Whitney *U* test was used if the data were still not normally distributed. Multivariate, forward stepwise linear regression analyses were used to identify predictors of bone microarchitecture and estimated bone strength. All the statistical computations were run using SPSS software, version 22.0 for Windows (SPSS Inc.), and *P* < 0.05 was considered to be statistically significant.

## Results

### Demographic, clinical and biochemical characteristics

General characteristics of CO AGHD patients and healthy male controls are shown in Table 1. A total of 20 male patients with CO AGHD were enrolled in this cross-sectional study. Fifteen patients had pituitary stalk interruption and four patients had pituitary hypoplasia based on MRI. Eleven of them (11/19) were born with breech or foot presentation. Another one patient underwent surgery for craniopharyngioma at the age of 4 years. All patients had MPHD. Fifteen patients sustained levothyroxine replacement therapy. Eight patients sustained glucocorticoid replacement. Testosterone replacement was started at 18 years old and after completion of linear growth in all patients. All patients accepted rhGH replacement therapy since childhood. Duration of rhGH replacement treatment was 11.2 ± 3.5 years. The average time course since the cessation of rhGH replacement was 6.6 ± 3.3 years. No fracture was reported from all our patients.

**Table 1:** Clinical and anthropometric characteristics of the study population. Data are presented as mean ± s.d.

	Control (*n* = 30)	CO AGHD (*n* = 20)	*P* value
Age (year)	30.6 ± 4.9	28.2 ± 5.4	0.104
Height (cm)	174.5 ± 5.3	167.1 ± 6.8	<0.001
Weight (kg)	70.7 ± 9.6	71.6 ± 11.0	0.744
Waist circumference (cm)	84.1 ± 6.7	95.1 ± 10.3	<0.001
Hip circumference (cm)	96.9 ± 6.4	98.2 ± 6.8	0.512
Waist-hip ratio	0.87 ± 0.05	0.96 ± 0.08	<0.001
FM (kg)	16.5 ± 5.5	19.4 ± 6.3	0.086
FM%	22.8 ± 4.9	27.0 ± 5.9	0.009
FFM (kg)	54.3 ± 5.4	51.3 ± 8.8	0.196
TBW (kg)	39.7 ± 3.9	37.6 ± 6.5	0.199
BMI (kg/m^2^)	23.2 ± 2.8	25.6 ± 3.7	0.014
FMI (kg/m^2^)	5.4 ± 1.7	7.0 ± 2.5	0.010
FFMI (kg/m^2^)	17.7 ± 1.4	18.3 ± 2.5	0.339
Grip strength of non-dominant hand (kg)	45.4 ± 7.9	37.0 ± 4.3	<0.001

BMI, body mass index; FFM, fat-free mass; FFMI, fat-free mass index; FM, fat mass; FMI, fat mass index; TBW, total body water.

There was no difference in chronologic age between both groups (28.2 ± 5.4 vs 30.6 ± 4.9 years, *P* = 0.104). Compared with the control group, CO AGHD group had a 7.4cm lag in final height (167.1 ± 6.8 vs 174.5 ± 5.3 cm, *P* < 0.001). CO AGHD group had increased BMI (25.6 ± 3.7 vs 23.2 ± 2.8, *P* = 0.014), an 11.0 cm increase in waist circumference (95.1 ± 10.3 vs 84.1 ± 6.7 cm, *P* < 0.001) and increased waist-hip ratio (0.96 ± 0.08 vs 0.87 ± 0.05, *P* < 0.001). AGHD group also had a 4.2% increase in fat mass percentage (27.0 ± 5.9 vs 22.8 ± 4.9%, *P* = 0.009) and increased FMI (7.0 ± 2.5 vs 5.4 ± 1.7, *P* = 0.010). No difference was found in FM, FFM or TBW between two groups. Besides, AGHD patients had an 8.4kg decrease in grip strength (37.3 ± 4.0 vs 45.7 ± 8.2 kg, *P* < 0.001) and a significantly decreased creatinine level (68.1 ± 14.8 vs 82.6 ± 9.1 μmol/L, *P* = 0.001).

Endocrinological and biochemical parameters are listed in Table 2. Patients with CO AGHD had a significantly decreased IGF-1 level (51.0 ± 29.9 vs 231.2 ± 66.9 ng/mL, *P* < 0.001) and IGF-1 SDS (−4.62 ± 2.07 vs 0.32 ± 0.80, *P* < 0.001). Before glucocorticoids replacement, CO AGHD group had a significant lower morning cortisol levels than controls (8.4 ± 5.6 vs 13.2 ± 4.0 μg/dL, *P* = 0.003). With monthly replacement of testosterone, CO AGHD patients had similar testosterone levels as control group (*P* = 0.334). With daily replacement with l-thyroxin, FT3 and FT4 levels in all CO AGHD patients were in the normal ranges but lower than normal controls (*P* < 0.001).

**Table 2:** Endocrinological and biochemical evaluation of the study population. Data are presented as mean ± s.d.

	Control (*n* = 30)	CO AGHD (*n* = 20)	*P* value
IGF-1 (ng/mL)	231.2 ± 66.9	51.0 ± 29.9	<0.001
IGF-1 SDS	0.32 ± 0.80	−4.62 ± 2.07	<0.001
ACTH (pg/mL)	28.2 ± 16.6	29.2 ± 14.8	0.057
Cortisol (μg/dL)	13.2 ± 4.0	8.4 ± 5.6	0.003
LH (IU/L)	3.69 ± 1.44	0.45 ± 0.66	<0.001
FSH (IU/L)	4.24 ± 1.76	0.88 ± 0.93	<0.001
T (ng/mL)	4.66 ± 1.35	5.08 ± 1.73	0.334
TSH (μIU/mL)	1.947 ± 0.810	1.875 ± 1.027	0.783
FT3 (pg/mL)	3.30 ± 0.33	2.61 ± 0.57	<0.001
FT4 (ng/dL)	1.39 ± 0.19	0.96 ± 0.25	<0.001
Glu (mmol/L)	5.29 ± 0.40	4.94 ± 0.47	0.006
HbA1c (%)	5.14 ± 0.25	5.30 ± 0.15	0.106
Hb (g/L)	155.6 ± 12.3	141.5 ± 12.1	<0.001
ALT (U/L)	26.2 ± 11.7	19.7 ± 9.7	0.038
AST(U/L)	21.6 ± 4.8	26.3 ± 8.6	0.005
ALP (U/L)	66.7 ± 13.3	148.7 ± 65.6	<0.001
Alb (g/L)	48.5 ± 2.0	47.3 ± 2.9	0.083
PA (mg/L)	301.3 ± 40.1	229.8 ± 69.6	<0.001
Tbil (μmol/L)	16.2 ± 6.3	12.8 ± 6.4	0.074
Dbil (μmol/L)	5.2 ± 2.1	4.2 ± 2.4	0.090
Ca (mmol/L)	2.36 ± 0.07	2.39 ± 0.07	0.115
P (mmol/L)	1.12 ± 0.12	1.43 ± 0.23	<0.001
25 OHD (ng/mL)	23.28 ± 8.03	21.58 ± 8.41	0.532
PTH (pg/mL)	41.63 ± 12.93	48.54 ± 25.95	0.275
β-CTX (ng/mL)	0.29 ± 0.13	0.72 ± 0.28	<0.001
Cr (μmol/L)	82.6 ± 9.1	68.1 ± 14.8	0.001
UA (μmol/L)	360.9 ± 69.3	380.9 ± 78.6	0.350

25 OHD, 25 hydroxyvitamin D; ACTH, adrenocorticotropic hormone; Alb, albumin; ALP, alkaline phosphatase; ALT, alanine transaminase; AST, aspartate aminotransferase; Ca, calcium; Cr, creatinine; Dbil, direct bilirubin; FSH, follicle stimulating hormone; FT3, free triiodothyronine; FT4, free thyroxine; Glu, glucose; Hb, hemoglobin; HbA1c, glycosylated hemoglobin; IGF-1, insulin like growth factor 1; LH, luteinizing hormone; P, phosphate; PA, prealbumin; PTH, parathyroid hormone; T, testosterone; Tbil, total bilirubin; TSH, thyroid stimulating hormone; UA, uric acid; β-CTX, β-C-terminal telopeptide region of collagen type 1.

There were no routine calcium pills or vitamin D intake in either group. Calcium levels were similar between the two groups. There was a significant increase in serum phosphate (1.43 ± 0.23 vs 1.12 ± 0.12 mmol/L, *P* < 0.001) and alkaline phosphatase levels (148.7 ± 65.6 vs 66.7 ± 13.3 U/L, *P* < 0.001) in CO AGHD group. Meanwhile, serum β-CTX levels in CO AGHD group also showed a significant increase (0.72 ± 0.28 vs 0.29 ± 0.13 ng/mL, *P* < 0.001), while there was no difference in serum levels of parathyroid hormone and 25 hydroxyvitamin D. There was no difference in other biochemical parameters including HbA1c, albumin, total bilirubin, direct bilirubin and uric acid levels between two groups.

### vBMD and microarchitecture of the distal tibia

Data obtained by HR-pQCT of the distal tibia was shown in Fig.1 and listed in Table 3. Compared with healthy controls, CO AGHD group had significantly decreased total vBMD (209.61 ± 45.73 vs 326.98 ± 66.87 mg HA/cm^3^, *P* < 0.001), as well as cortical vBMD (850.53 ± 53.21 vs 929.51 ± 37.08 mg HA/cm^3^, *P* < 0.001) and trabecular vBMD (136.38 ± 32.76 vs 188.86 ± 42.20 mg HA/cm^3^, *P* < 0.001) in distal tibia.

**Table 3:** HR-pQCT parameters of the distal tibia in CO AGHD group and controls. Data are presented as mean ± s.d.

	Control (*n* = 30)	CO AGHD (*n* = 20)	*P* value
Total area (mm^2^)	801.49 ± 116.45	933.37 ± 152.78	0.001
Total vBMD (mg HA/cm^3^)	326.98 ± 66.87	209.61 ± 45.73	<0.001
Cortical area (mm^2^)	150.09 ± 28.17	95.92 ± 20.22	<0.001
Cortical vBMD (mg HA/cm^3^)	929.51 ± 37.08	850.53 ± 53.21	<0.001
Cortical perimeter (mm)	110.24 ± 8.09	118.65 ± 10.39	0.002
Cortical thickness (mm)	1.591 ± 0.365	0.910 ± 0.204	<0.001
Intra-cortical porosity	0.023 ± 0.013	0.010 ± 0.004	<0.001
Trabecular area (mm^2^)	657.14 ± 124.23	843.65 ± 152.51	<0.001
Trabecular vBMD (mg HA/cm^3^)	188.86 ± 42.20	136.38 ± 32.76	<0.001
Trabecular thickness (mm)	0.267 ± 0.026	0.236 ± 0.021	<0.001
Trabecular number (1/mm)	1.378 ± 0.177	1.331 ± 0.253	0.449
Trabecular separation (mm)	0.710 ± 0.095	0.754 ± 0.186	0.501
Tb.1/N.SD (mm)	0.297 ± 0.043	0.300 ± 0.096	0.235
Trabecular bone volume fraction	0.282 ± 0.054	0.213 ± 0.046	<0.001
Bone stiffness (N/mm)	239 248.1 ± 43 866.4	165 377.8 ± 41 389.1	<0.001
Bone failure load (N)	12 889.1 ± 2191.3	9151.1 ± 2197.9	<0.001

Tb.1/N.SD, St.Dev of 1/Tb.N, inhomogeneity of network; vBMD, volumetric bone mineral density.

CO AGHD group also had significant decreased cortical area (95.92 ± 20.22 vs 150.09 ± 28.17 mm^2^, *P* < 0.001), cortical thickness (0.910 ± 0.204 vs 1.591 ± 0.365 mm, *P* < 0.001), intra-cortical porosity (0.010 ± 0.004 vs 0.023 ± 0.013, *P* < 0.001), trabecular thickness (0.236 ± 0.021 vs 0.267 ± 0.026 mm, *P* < 0.001) and trabecular bone volume fraction (0.213 ± 0.046 vs 0.282 ± 0.054, *P* < 0.001) in distal tibia.

At the same time, CO AGHD group had significant increased total area (933.37 ± 152.78 vs 801.49 ± 116.45 mm^2^, *P* = 0.001), cortical perimeter (118.65 ± 10.39 vs 110.24 ± 8.09 mm, *P* = 0.002) and trabecular area (843.65 ± 152.51 vs 657.14 ± 124.23 mm^2^, *P* < 0.001) in distal tibia.

### vBMD and microarchitecture of the radius

Data obtained by HR-pQCT of the radius was shown in Fig.1 and listed in Table 4. Compared with healthy controls, AGHD group had significantly decreased total vBMD (226.38 ± 56.90 vs 355.35 ± 66.49 mg HA/cm^3^, *P* < 0.001), as well as cortical vBMD (747.16 ± 80.22 vs 919.51 ± 38.68 mg HA/cm^3^, *P* < 0.001) and trabecular vBMD (140.92 ± 37.60 vs 184.65 ± 39.82 mg HA/cm^3^, *P* < 0.001) in radius.

**Table 4:** HR-pQCT parameters of the distal radius in CO AGHD group and controls. Data are presented as mean ± s.d.

	Control (*n* = 30)	CO AGHD (*n* = 20)	*P* value
Total area (mm^2^)	324.96 ± 54.26	343.54 ± 71.24	0.301
Total vBMD (mg HA/cm^3^)	355.35 ± 66.49	226.38 ± 56.90	<0.001
Cortical area (mm^2^)	75.54 ± 10.74	47.60 ± 9.82	<0.001
Cortical vBMD (mg HA/cm^3^)	919.51 ± 38.68	747.16 ± 80.22	<0.001
Cortical perimeter (mm)	75.65 ± 6.56	77.77 ± 9.87	0.406
Cortial thickness (mm)	1.186 ± 0.193	0.706 ± 0.195	<0.001
Intra-cortical porosity	0.006 ± 0.004	0.004 ± 0.002	0.126
Trabecular area (mm^2^)	253.48 ± 55.27	300.12 ± 72.64	0.013
Trabecular vBMD (mg HA/cm^3^)	184.65 ± 39.82	140.92 ± 37.60	<0.001
Trabecular thickness (mm)	0.246 ± 0.018	0.215 ± 0.018	<0.001
Trabecular number (1/mm)	1.462 ± 0.180	1.525 ± 0.236	0.294
Trabecular separation (mm)	0.633 ± 0.083	0.641 ± 0.116	0.756
Tb.1/N.SD (mm)	0.250 ± 0.036	0.246 ± 0.056	0.810
Trabecular bone volume fraction	0.273 ± 0.056	0.203 ± 0.054	<0.001
Bone stiffness (N/mm)	89 036.9 ± 17 207.6	47 397.1 ± 15 741.4	<0.001
Bone failure load (N)	4839.2 ± 905.3	2639.9 ± 889.4	<0.001

Tb.1/N.SD, St.Dev of 1/Tb.N, inhomogeneity of network; vBMD, volumetric bone mineral density.

Compared with healthy controls, AGHD group had significant decrease in cortical area (47.60 ± 9.82 vs 75.54 ± 10.74 mm^2^, *P* < 0.001), cortical thickness (0.706 ± 0.195 vs 1.186 ± 0.193 mm, *P* < 0.001), trabecular thickness (0.215 ± 0.018 vs 0.246 ± 0.018 mm, *P* < 0.001) and trabecular bone volume fraction (0.203 ± 0.054 vs 0.273 ± 0.056, *P* < 0.001). Trabecular area (300.12 ± 72.64 vs 253.48 ± 55.27 mm^2^, *P* = 0.013) in AGHD group was significantly increased in AGHD group.

### Finite element analysis

At the tibia (Table 3), bone stiffness (165 377.8 ± 41 389.1 vs 239 248.1 ± 43 866.4, *P* < 0.001) and failure load (9151.1 ± 2197.9 vs 12 889.1 ± 2191.3, *P* < 0.001) of the AGHD group were significantly decreased when compared with the control group. Similarly, at the radius (Table 4), bone stiffness (47 397.1 ± 15 741.4 vs 89 036.9 ± 17 207.6, *P* < 0.001) and failure load (2639.9 ± 889.4 vs 4839.2 ± 905.3, *P* < 0.001) of the AGHD group were also significantly decreased in comparison with the healthy controls.

### Association between IGF-1 levels and parameters of bone microarchitecture and estimated bone strength

On multivariate analysis, after adjusting for age, BMI and serum levels of testosterone and FT4, serum IGF-1 level was a positive predictor for total vBMD (β = 0.4710, *P* < 0.001), cortical vBMD (β = 0.3087, *P* < 0.001), cortical area (β = 0.2347, *P* < 0.001), cortical thickness (β = 0.0029, *P* < 0.001), trabecular vBMD (β = 0.2030, *P* = 0.001), trabecular thickness (β = 0.0001, *P* = 0.001), trabecular bone volume fraction (β = 0.0003, *P* = 0.001), intra-cortical porosity (β = 0.00005, *P* = 0.004), bone stiffness (β = 315.14, *P* < 0.001) and failure load (β = 15.92, *P* < 0.001) of the distal tibia.Table 5

**Table 5:** Associations between bone microarchitecture of the distal tibia and IGF-1 levels after adjusting for age, BMI and serum levels of testosterone and FT4.

	β	*P* value
Total vBMD (mg HA/cm^3^)	0.4710	<0.001
Cortical area (mm^2^)	0.2347	<0.001
Cortical vBMD (mg HA/cm^3^)	0.3087	<0.001
Cortical thickness (mm)	0.0029	<0.001
Intra-cortical porosity	0.00005	0.004
Trabecular vBMD (mg HA/cm^3^)	0.2030	0.001
Trabecular thickness (mm)	0.0001	0.001
Trabecular bone volume fraction	0.0003	0.001
Bone stiffness (N/mm)	315.14	<0.001
Bone failure load (N)	15.92	<0.001

vBMD, volumetric bone mineral density.

**Table 6:** Associations between bone microarchitecture of the distal radius and IGF-1 levels after adjusting for age, BMI and serum levels of testosterone and FT4.

	β	*P* value
Total vBMD (mg HA/cm^3^)	0.4781	<0.001
Cortical area (mm^2^)	0.1096	<0.001
Cortical vBMD (mg HA/cm^3^)	0.6503	<0.001
Cortical thickness (mm)	0.0019	<0.001
Trabecular area (mm^2^)	−0.1797	0.046
Trabecular vBMD (mg HA/cm^3^)	0.1565	0.008
Trabecular thickness (mm)	0.0001	<0.001
Trabecular bone volume fraction	0.0003	0.003
Bone stiffness (N/mm)	161.25	<0.001
Bone failure load (N)	8.49	<0.001

vBMD, volumetric bone mineral density.

As for the radius, after adjusting for age, BMI and serum levels of testosterone and FT4, serum IGF-1 level was a positive predictor for total vBMD (β = 0.4781, *P* < 0.001), cortical vBMD (β = 0.6503, *P* < 0.001), cortical area (β = 0.1096, *P* < 0.001), cortical thickness (β = 0.0019, *P* < 0.001), trabecular vBMD (β = 0.1565, *P* = 0.008), trabecular thickness (β = 0.0001, *P* < 0.001), trabecular bone volume fraction (β = 0.0003, *P* = 0.003), bone stiffness (β = 161.25, *P* < 0.001) and failure load (β = 8.49, *P* < 0.001). Serum IGF-1 level was also a negative predictor for trabecular area (β = −0.1797,* P* = 0.046) of the radius (Table 6).

## Discussion

In this study, we focused on vBMD, bone microarchitecture and estimated bone strength of young male adult with CO AGHD and compared with age-matched controls. Our results showed that (1) vBMD of both distal tibia and non-dominant radius were significantly decreased in AGHD patients; (2) CO AGHD patients had significantly decreased cortical area and cortical thickness, as well as trabecular thickness and trabecular bone volume fraction of both tibia and distal radius; (3) CO AGHD patients had lower estimated bone strength; (4) after adjusting for age, BMI and serum levels of testosterone and free T4, serum IGF-1 level was a positive predictor for total vBMD, cortical vBMD, cortical area, trabecular vBMD, bone stiffness and failure load. In our series of CO AGHD patients, the average time course since cessation of rhGH replacement was 6.6 ± 3.3 years and all patients sustained testosterone replacement since 18 years old. We thus conclude that young adult male patients with CO AGHD who are no longer receiving GH replacement have abnormalities in bone microarchitecture and estimated bone strength.

Low bone mass is widely believed as a characteristic feature of AGHD since the GH/IGF axis plays a pivotal role in skeletal growth and strength. GH activates both bone formation and resorption via direct and indirect mechanisms (19). IGF-1 regulates radial bone growth and cortical and trabecular bone properties via their effects on osteoblast, osteocyte and osteoclast function (20). Both bone size and BMD increase gradually throughout childhood and rapidly increase during puberty. Calcium is added to bone most rapidly since about age 9 in girls and age 10 in boys (21). During puberty, interactions of IGF-1 and gonadosteroids and parathyroid hormone contribute to the appropriate accumulation of bone mass. Thus, BMD continues to increase until age reaches the mid-to-late 20s and decline gradually throughout the rest of adult life.

However, there were lots of inconsistencies of data of bone density and bone microarchitecture features in AGHD patients. Data from Stephen *et al*. described normal trabecular bone mineral density and only a 2% decrease in cortical density in radius was described in 13 CO AGHD patients (22). It was suggested that the apparent low BMD observed with DXA was a reduction in cortical bone volume and not density. In our series of patients, rhGH was stopped at the end of puberty and testosterone replacement was sustained in early adulthood. vBMD of both distal tibia and non-dominant radius were significantly decreased. At the same time, the increased β-CTX and ALP suggested active turnover of bone in AGHD patients. While serum levels of both 25 OHD and PTH were all in normal ranges, which suggested that the low vBMD and impaired bone microarchitecture were mainly caused by low IGF-1 levels. As we know, peak bone mass is reached after final adult height, at a mean of 23.1 years in males and 19.9 years in females (23). There was a concurrence of formative years of peak bone development and cessation of rhGH replacement. After adjusting for age, BMI and serum testosterone levels, serum IGF-1 level was a positive predictor for total vBMD, cortical vBMD, cortical area and trabecular vBMD of both tibia and distal radius. Our work thus shed new insight into the seamless transition of childhood-onset GHD from pediatric to teenage and young adulthood.

Our results also provide new insight into the clinical characteristics of AGHD with different age of onset. AGHD is a group of condition with heterogeneous etiologies. There were striking regional variations in pathogenesis and clinical characteristics among adult-onset AGHD (AO AGHD) in Europe and the United States (24). In China, AGHD is less recognized and treated comparing with Western countries and no consensus of management has been reached so far (25). A proportion of patients with childhood-onset GHD are idiopathic and the growth hormone insufficiency is transient (26). While in patients with MPHD, GHD is usually permanent (27). A large number of patients with MPHD drop out of rhGH replacement therapy during the transition period. There are a lot of associated factors, including insufficient knowledge of treatment necessity in patients, their family and health providers (8), inconvenience of rhGH administration, as well as social-economic issues. In recent data from 16 AO AGHD patients due to sellar masses or traumatic brain injury (8), bone microarchitecture was not deficient in these patients. Our data thus supported that CO AGHD due to MPHD is a distinct entity in AGHD and should be treated in different way from AO AGHD.

The main limitation of our study is the small number of participants. But there was good homogeneity in the etiology, age of onset, duration of rhGH replacement therapy and time course since cessation of rhGH treatment. Inconsistency of etiology and disease duration is one of the main reasons for the inconsistently reported clinical characteristics. Another limitation is the cross-sectional nature of our study. We are going to follow-up these patients in long term and evaluate the dynamic changes of bone microarchitecture and risk of fracture in the future. At the same time, further studies to evaluate effects of rhGH replacement therapy on vBMD and microstructure of CO AGHD patients due to MPHD are needed to elucidate the cause–effect relationship between IGF-1 levels and bone quality. The third limitation is that no questionnaire about exercise was carried out in this study, since appropriate exercise is capable of increasing bone mass and strength.

In conclusion, our findings indicate that CO AGHD patients who are no longer receiving growth hormone replacement had significantly impaired vBMD and microarchitecture and lower estimated bone strength in radius and distal tibia. IGF-1 level is positive predictor for total vBMD, cortical vBMD, cortical area, trabecular vBMD, bone stiffness and failure load. Seamless transition of management is advocated in childhood-onset AGHD patients due to MPHD.

## Declaration of interest

The authors declare that there is no conflict of interest that could be perceived as prejudicing the impartiality of this study.

## Funding

This work was supported by the National Science Foundation of China (No. 81400774), PUMCH Youth Fund (No. 3332016128) and the Special Research Fund for Central Universities, Peking Union Medical College (No. 2017PT31004) for Hongbo Yang.

## Author contribution statement

H Y designed the study and wrote the primary manuscript. H Z and H P designed and supervised the study and revised the primary manuscript. K Y took part in the collection of clinical data and analyzed the data. Q Z performed the biochemical measurements. L W contributed to the study management. F G helped to revise the primary manuscript.
